# Non-translational Connections of eEF1B in the Cytoplasm and Nucleus of Cancer Cells

**DOI:** 10.3389/fmolb.2020.00056

**Published:** 2020-04-09

**Authors:** Boris Negrutskii

**Affiliations:** Department of Structural and Functional Proteomics, Institute of Molecular Biology and Genetics, National Academy of Sciences, Kyiv, Ukraine

**Keywords:** supramolecular complex, translation, elongation factors, eEF1B, intracellular localization, cancer, cytoplasm, nucleus

## Abstract

The human translation machinery includes three types of supramolecular complexes involved in elongation of the polypeptide chain: the ribosome, complex of elongation factors eEF1B and multienzyme aminoacyl-tRNA synthetase complex. Of the above, eEF1B is the least investigated assembly. Recently, a number of studies provided some insights into the structure of different eEF1B subunits and changes in their expression in cancer and other diseases. There is increasing agreement that possible disease-related functions of eEF1B are not necessarily related to its role in translation. This mini-review focuses on structural and functional features of the eEF1B complex while paying special attention to possible non-canonical functions of its subunits in cancer cells.

## Introduction

Several comprehensive reviews describing the basics of eEF1B structure and function have been published (see, for example, Le Sourd et al., 2006; Sasikumar et al., 2012). This mini-review summarizes novel structural, functional and expression data and presents recent information about possible cancer-related links of eEF1B subunits. Special attention is paid to newly emerging questions such as how stable the complex is in cancer tissues and how non-canonical involvements of its individual subunits are related to their intracellular localization. I regret that some important features of eEF1B remained undescribed due to space constrains.

### Structural Insights

From the moment of its discovery, the eEF1B complex was considered as a stable unit which is essential for peptide elongation on the 80S ribosome. The human complex comprises three subunits: eEF1Bα (EEF1B2), eEF1Bβ (EEF1D), and eEF1Bγ (EEF1G), according to established nomenclature (IUBMB, 1996).

eEF1Bα is a non-globular protein which shows a noticeably extended shape in solution (Trosiuk et al., 2016). The C-terminal part of eEF1Bα executes nucleotide exchange while the N-terminal fragment connects the protein with the eEF1Bγ subunit. The N-terminal part of eEF1Bα plays an autoinhibitory role, while the presence of eEF1Bγ stimulates eEF1Bα-mediated nucleotide exchange (Trosiuk et al., 2016). The NMR-derived structure of the part of the eEF1Bα C-terminal domain is known (Pérez et al., 1999).

eEF1Bβ and eEF1Bα have similar domain structure. The C-terminal part of eEF1Bβ is highly homologous to the C-terminal domain of eEF1Bα, while the N-terminal fragment has the unique amino acid sequence, a part of which is a leucine-zipper structure responsible for oligomerization of eEF1Bβ (Bondarchuk et al., 2016). The very N-terminal fragment (1-77 amino acid residues) binds eEF1Bγ and, possibly, other proteins (Bondarchuk et al., 2019), however, we cannot exclude at present that the remaining part of the eEF1Bβ N-terminal domain is also involved in eEF1Bγ binding. This domain shows a dynamic spatial organization provided by α-helical segments linked by flexible linkers (Bondarchuk et al., 2019). The NMR-derived structure of the eEF1Bβ C-terminal fragment is known (Wu et al., 2016).

eEF1Bγ has no nucleotide exchange activity while playing instead a scaffold role in the complex as it hosts eEF1Bα and eEF1Bβ. Structural investigation of this subunit is complicated by its high tendency to aggregate (Sheu and Traugh, 1997, Kamiie et al., 2003). The NMR-derived structure of the 19 kDa C-terminal fragment of human protein (Vanwetswinkel et al., 2003) and crystal structure of the N-terminal domain of yeast protein (Jeppesen et al., 2003) are known.

Of note, unpublished structures of the complex of the eEF1Bα N-terminal domain and eEF1Bγ N-terminal domain and of the complex of the eEF1Bγ N-terminal domain and eEF1Bβ N-terminal domain are available in PDB (5DQS and 5JPO, correspondingly).

### Functional Insights

#### Translation Functions

To date, the only established function of the eEF1B complex is to catalyze nucleotide exchange in another elongation factor, eEF1A, which supplies the ribosomes with aminoacyl-tRNAs. Both eEF1Bα and eEF1Bβ possess the nucleotide exchange activity. Importantly, different tissue-specific isoforms of eEF1A showed different eEF1Bα-dependence *in vitro*, namely, eEF1A2 rather than eEF1A1 strongly needed eEF1Bα for nucleotide exchange (Trosiuk et al., 2016). No corresponding information about eEF1Bβ is available. Deletion of the yeast eEF1Bγ gene induced increased misreading of near-cognate codons and decreased misreading of non-cognate codons (Plant et al., 2007). Interestingly, the yeast eEF1B complex was suggested to serve as an exchange agent for translation termination factor eRF3 (Valouev et al., 2009).

#### Non-canonical Functions

Beyond translation, subunits of eEF1B may be involved in many different processes, including cell cycle control, cytoskeleton regulation, and stress response, as summarized in Sasikumar et al. (2012). This section is focused on new non-canonical links of eEF1B revealed by a proteomic approach. Post-translational modification-related regulation of eEF1B subunits is not described here in full due to space constrains.

##### eEF1Bα

There is not too much data on non-canonical functions of eEF1Bα. This protein interacts with nuclear/cytoplasmic prolyl-isomerase FKBP25 (FKBP3) (Galat et al., 2014), which is a nucleic acid binding protein involved in both repair of DNA double-strand breaks (Dilworth et al., 2019) and microtubule polymerization (Dilworth et al., 2018). Depletion of nucleoside diphosphate kinase A (NME1) lead to a redistribution of eEF1Bα from the cytoplasm to the endoplasmic reticulum fraction in mouse liver (Bruneel et al., 2011). Importantly, nucleoside diphosphate kinase A can be transferred to the nucleus where it promotes the non-homologous end joining of the DNA double-strand breaks (Xue et al., 2019) and takes part in transcription (Puts et al., 2018).

The N-terminal fragment of eEF1Bα was reportedly secreted from mouse astrocytes upon stimulation of a Ca^2+^ increase by bradykinin treatment (Yin et al., 2012) though there was no explanation for the phenomenon.

##### eEF1Bβ

Some non-canonical involvements of eEF1Bβ in cell functioning are predicted. eEF1Bβ is a potential substrate of sucrose non-fermenting related kinase (SNRK) which controls adipose inflammation and energy homeostasis (Li et al., 2018). Importantly, knocking down eEF1Bβ expression in adipocytes induced inflammation (Li et al., 2018). Decreased expression of eEF1Bβ was detected during inflammation in lipid A-treated mouse epithelial cells (Takano et al., 2016).

eEF1Bβ inhibited auto-ubiquitination and degradation of E3 ubiquitin ligase (SIAH-1) (Wu et al., 2011). There was 3-fold increase in the amount of eEF1Bβ in pancreas of diabetic rats (Jiang et al., 2011). Upregulation of eEF1Bβ was reported in lungs of chronic hypoxic rats with pulmonary hypertension. Peculiarly, eEF1Bβ was heavily stained in the apical region of the epithelium in bronchioles, as well as in lymphocytes and macrophages interstitially dispersed in the epithelium (Østergaard et al., 2011).

eEF1Bβ was markedly decreased in senescent human fetal lung diploid fibroblasts cells, while treatment of these cells with a putative anti-aging agent caused an opposite effect (Xing et al., 2018). Similar influence of senescence on eEF1Bα (Byun et al., 2009) and eEF1Bβ (Takano et al., 2016), as well as on eEF1A1 (Byun et al., 2009) apparently reflects the global inhibition of protein synthesis during aging which supposedly occurs due to suppression of the whole eEF1B complex. Thus, an effect of senescence on eEF1B canonical function is also possible.

##### eEF1Bγ

The eEF1Bγ subunit shows a large variety of prospective non-canonical functions. A high in unsaturated fatty acids diet during gestation and lactation caused more than 5-fold eEF1Bγ increase in neonatal offspring liver in rats (Novak et al., 2009). eEF1Bγ was decreased in human myometrium after exogenous oxytocin administration while a reciprocal effect was observed upon administration of an oxytocin antagonist (de Wit et al., 2010).

Yeast eEF1Bγ and not the eEF1Bγ-eEF1Bα complex were supposedly involved to the retrograde Golgi to ER transport (Esposito and Kinzy, 2010). In other fungi, *Aspergillus fumigatus*, deletion of eEF1Bγ was associated with changes in oxidative stress and actin cytoskeleton organization (O’Keeffe et al., 2013). The gills from Atlantic halibut exposed to water containing high CO_2_ concentration showed upregulation of eEF1Bγ (Bresolin de Souza et al., 2014).

eEF1Bγ was one of the candidates to participate in loading of microRNAs into extracellular vesicles that may be needed to preserve microRNAs and transfer them to extracellular fluid (Hagiwara et al., 2015). eEF1Bγ interacted with components of DNA damage response machinery (Pisani et al., 2016). Yeast eEF1Bγ was found in stress granules upon heat shock (Grousl et al., 2013; Cherkasov et al., 2015). Interestingly, upon heat stress of yeast, all subunits of the eEF1B complex aggregate quickly and synchronously, contrary to eEF1A (Wallace et al., 2015). eEF1Bγ was identified as a member of the pre-mRNA 3′ end cleavage complex (Shi et al., 2009).

Oncogene-induced senescence in primary human fibroblasts caused a marked (more than 9-fold) increase in ubiquitination of eEF1Bγ. Peculiarly, no changes in ubiquitination of other subunits of eEF1B were found, while eEF1A, several aminoacyl-tRNA synthetases and translation initiation factors were also hyper ubiqiutinated (Bengsch et al., 2015). It should be noted, however, that mono- and polyubiquitination of eEF1Bγ were not differentiated in this case. eEF1Bγ was reported to positively affect K63-linked polyubiquitination of mitochondrial antiviral-signaling protein (MAVS) and negatively influence its K48-linked polyubiquitination resulting in the increased abundance of this antiviral protein (Liu et al., 2014).

An EEF1G– anaplastic lymphoma kinase (ALK) gene fusion was recently found in two pediatric patients with anaplastic large cell lymphoma (Palacios et al., 2017). In the context of the review, it is important that a fusion of the eEF1Bγ N-terminal domain provided exclusively cytoplasmic localization of the fusion protein (Ceccon et al., 2016) suggesting that the N-terminal domain of eEF1Bγ could not possibly be related to nuclear localization of eEF1Bγ alone.

Drosophila eEF1Bγ is a substrate of LAMMER kinase (DOA) and can be phosphorylated by human kinase LAMMER/CLK2 (CLK2) as well (Fan et al., 2010). Importantly, DOA kinase and eEF1Bγ negatively regulated motor-mediated transport of membrane organelles along microtubules depending on the phosphorylation of Ser 294 in eEF1Bγ (Serpinskaya et al., 2014).

### eEF1B Proteins in Cancer

The levels of mRNA and a protein coded by this mRNA do not often correlate (Liu et al., 2016) which is valid for eEF1B subunits as well (Veremieva et al., 2011, 2014; Flores et al., 2016). This mini-review is focused on eEF1B proteins, therefore cancer-related changes in the levels of corresponding mRNAs are not considered here, although such changes may be of marker/prognostic importance (Hassan et al., 2018; Biterge-Sut, 2019). It is noteworthy that the data described in this section may be related to both canonical and non-canonical functioning of eEF1B subunits.

Not too much information is available on eEF1Bα association with cancer. Increased expression of eEF1Bα was reported as a negative prognosis marker in stomach cancer (Jia et al., 2018). Besides, ionizing radiation-induced senescence of breast cancer, lung cancer and colon cancer cell lines was accompanied by a significant decrease in eEF1Bα (Byun et al., 2009).

eEF1Bβ was long known as a cadmium-induced proto-oncogene (Joseph et al., 2002; Lei et al., 2002; Lei et al., 2010). Recently, eEF1Bβ was proposed as a blood marker for cadmium exposure (Lu et al., 2013). This protein was upregulated in the metastatic canine mammary carcinoma (Klopfleisch et al., 2010) and human oral squamous cell carcinoma (Flores et al., 2016) tissues, as well as in 5-fluorouracil-resistant human breast cancer cells (Zheng et al., 2010). Interestingly, knocking down of eEF1Bβ mRNA was accompanied by the opposite effects on the proliferation and epithelial mesenchymal transition (EMT)/invasion capabilities of squamous cell carcinoma cells (Flores et al., 2016). eEF1Bβ was upregulated in osteosarcoma and suggested to play a tumor-promoting role by facilitating the Akt-mTOR and Akt-Bad signaling pathways (Cheng et al., 2018).

The level of eEF1Bγ was increased in oral squamous cell carcinoma (Flores et al., 2016) and hepatic metastatic colorectal cancer (Yang et al., 2019). eEF1Bγ was downregulated in HepG2 cells treated with Nomura’s jellyfish venom used as an anti-cancer drug (Choudhary et al., 2018). Also, the protein was essentially decreased in cervical cancer specimens taken from patients after treatment with paclitaxel and cisplatin (Liu et al., 2011). eEF1Bγ may be an urine marker of bladder cancer (Chen et al., 2009). Interestingly, eEF1Bγ was 3-fold decreased in benign pleomorphic adenoma of the human parotid gland (Mutlu et al., 2017).

### Complex Matter

One may notice that cancer-related changes of individual eEF1B subunits were observed far more often than corresponding changes of all subunits together. It raised a question whether malignant tumors induce deregulation of individual subunits rather than the whole eEF1B complex?

An attempt to meet this challenge was undertaken in series of papers where cancer-related changes in the level of all three eEF1B subunits were analyzed simultaneously in the same tissue samples (Veremieva et al., 2011, 2014). More than two-fold upregulation of at least one eEF1B subunit was observed in 72% of 25 cardioesophageal cancer samples. The simultaneous increase in the level of all three eEF1B subunits was observed in the one, simultaneous upregulation of eEF1Bα and eEF1Bβ was observed in two cases and simultaneous upregulation of eEF1Bβ and eEF1Bγ was found in four cases while no correlated increase of eEF1Bα and eEF1Bγ was detected (Veremieva et al., 2011). More than two-fold rise in the level of at least one eEF1B subunit was observed in 52% of 25 lung carcinoma samples. Again, the simultaneous increase in the level of all three eEF1B subunits was observed in one case, simultaneous upregulation of eEF1Bα and eEF1Bβ was also found in one case, simultaneous upregulation of eEF1Bβ and eEF1Bγ was observed in five cases while no correlated elevation of eEF1Bα and eEF1Bγ was found (Veremieva et al., 2014). The authors concluded that cancer-related upregulation of individual subunits rather than the whole eEF1B complex takes place in both cancers. Interestingly, the observed trend of coordinated up-regulation of eEF1Bβ and eEF1Bγ was spotted also in oral squamous cell carcinoma (Flores et al., 2016). It is interesting that eEF1Bβ and eEF1Bγ showed also a parallel increase in the prenatal as compared to postnatal expression levels in mice brain and liver (Cao et al., 2014). Further experiments should reveal if these correlative changes reflect the formation *in vivo* of a stable complex eEF1Bβ-eEF1Bγ.

### Are Non-Canonical Functions of eEF1B Subunits Associated With Their Intracellular Localization?

Non-canonical functioning of eEF1B and/or its subunits is not yet attributed to specific cellular compartments. As translation occurs in the cytoplasm then the cytoplasmic localization of eEF1B is obvious. However, signs of the nuclear localization of eEF1B subunits were displayed in the normal (Drosophila embryos, human cardioesophageal junction, human lung) and cancer (cardioesophageal carcinoma, lung cancer, oral squamous cell carcinoma) tissues as well as in lung adenocarcinoma cells A549 (Fan et al., 2010; Veremieva et al., 2011; Veremieva et al., 2014; Flores et al., 2016). In an attempt to attribute possible non-canonical eEF1B functions to the specific cellular compartments the partners of eEF1Bβ and eEF1Bγ in the cytoplasm and nucleus of human lung carcinoma cells were identified experimentally and analyzed by several bioinformatics approaches (Kapustian et al., 2016, 2017, 2018, 2019). Cytoplasmic eEF1Bβ was predicted to be involved into four protein networks representing cell cycle regulation, DNA replication and repair, chromatin remodeling and chaperoning machinery (Kapustian et al., 2016). Nuclear eEF1Bβ interacted with proteins involved into RNA transcription and splicing, microRNA turnover, degradation of mRNA and proteins, DNA damage response. It could be also involved into adipose tissue biology and EMT (Kapustian et al., 2017). Cytoplasmic eEF1Bγ could participate in mRNA splicing and processing, nucleosome remodeling, cell cycle regulation, viral RNA transcription, oxidative stress response (Kapustian et al., 2018). Nuclear eEF1Bγ supposedly took part in splicing of pre-mRNA and regulation of mRNA stability, cytoskeleton-membrane linking and cellular trafficking (Kapustian et al., 2019).

One should mention that the co-immunoprecipitation data are not sufficient to provide an explicit evidence for an involvement of eEF1B subunits to one or the other process. However, analysis of these data is significant in that it provides a ground to develop the future direction of eEF1B research.

The Table 1 summarizes potential involvements of differently located eEF1B subunits in different cellular processes. No exclusive function can be attributed to cytoplasmic eEF1Bβ, while the only nucleus-localized eEF1Bβ may take part in adipose tissue biology (Kapustian et al., 2017). Both cytoplasmic and nuclear eEF1Bβ may be involved in EMT (Kapustian et al., 2016, 2017). The only cytoplasmic eEF1Bγ may participate in oxidative stress response (Kapustian et al., 2018). Taking into account the above-mentioned trends toward the coordinated upregulation of eEF1Bβ and eEF1Bγ in human cancer tissues it would be interesting to decipher the processes in which these subunits may be involved in concert. For the cytoplasmic fraction, those are, surprisingly, chromatin/nucleosome remodeling and cell cycle regulation (Kapustian et al., 2016, 2018) while for the nuclear fraction it is regulation of the mRNA stability and degradation (Kapustian et al., 2017, 2019). One should mention, however, that mixed intracellular localization is characteristic for components of these processes in cell. Besides, a number of processes may involve both cyto- and nucleo-localized eEF1Bβ and eEF1Bγ; these are microRNA turnover, cytoskeleton-membrane linking, cellular trafficking, mRNA transcription and splicing (Kapustian et al., 2016, 2017, 2018, 2019). This apparently reflects cyto-nucleo shuttling of subunits involved into these processes.

**TABLE 1:** Possible non-translational involvements of eEF1B subunits.

	cyto eEF1Bβ	nuclear eEF1Bβ	cyto eEF1Bγ	nuclear eEF1Bγ	eEF1Bα^#^
Chaperoning	Kapustian et al., 2016^+^				
MicroRNA turnover		Kapustian et al., 2017^+^	Hagiwara et al., 2015^+^*.		
Adipose tissue biology		Kapustian et al., 2017^+^; Li et al., 2018^+^*			
EMT	Kapustian et al., 2016^+^	Flores et al., 2016^+^*; Kapustian et al., 2017^+^			
Oxidative stress response			O’Keeffe et al., 2013^+^*; Kapustian et al., 2018^+^		
Cytoskeleton-membrane link	Ong et al., 2003^+^*			Kapustian et al., 2019^+^	
Cellular trafficking	Kapustian et al., 2016^+^			Serpinskaya et al., 2014^+^*; Kapustian et al., 2019^+^	
Splicing of mRNA		Kapustian et al., 2017^+^	Kapustian et al., 2018^+^	Fan et al., 2010; Kapustian et al., 2019^+^	
DNA repair	Kapustian et al., 2016^+^	Kapustian et al., 2017^+^			Bruneel et al., 2011; Galat et al., 2014
Chromatin and nucleosome remodeling	Kapustian et al., 2016^+^		Kapustian et al., 2018^+^		
Cell cycle regulation	Flores et al., 2016^+^*; Kapustian et al., 2016^+^		Kapustian et al., 2018^+^		
Transcription		Kapustian et al., 2017^+^	Kapustian et al., 2018^+^		
Stability and degradation of mRNA		Kapustian et al., 2017^+^		Kapustian et al., 2019^+^	
Microtubule interaction	Ong et al., 2003^+^*		Janssen and Möller, 1988^+^*		Galat et al., 2014
Actin cytoskeleton interaction			Furukawa et al., 2001^+^*		Furukawa et al., 2001
Intermediate filaments interaction			Kim et al., 2007^+^		
Ubiquitination	Wu et al., 2011^+^*		Liu et al., 2014; Bengsch et al., 2015^+^*		
Senescence	Takano et al., 2016; Xing et al., 2018 ^+^*				Byun et al., 2009

^+^Cytoplasmic or nuclear localization is defined. ^+*^Cytoplasmic or nuclear localization is not defined. ^#^Intracellular localization is not studied.

DNA repair may implicate both eEF1Bβ and eEF1Bα(Bruneel et al., 2011; Galat et al., 2014; Kapustian et al., 2016; Kapustian et al., 2017). This suggests an involvement of the whole eEF1B complex, as these subunits interact with each other via eEF1Bγ.

The viral infection-related functions of the eEF1B complex well described in Li et al. (2013) are not a subject of this mini-review, though some virus-related partners of eEF1B subunits were found in the cytoplasmic and nuclear fractions of lung carcinoma cells (Kapustian et al., 2017; Kapustian et al., 2018; Kapustian et al., 2019).

Surprisingly, a number of potential protein partners of cytoplasmic eEF1Bγ were linked to corneal diseases, particularly retinoblastoma (Kapustian et al., 2018; Kapustian et al., 2019). On the contrary, several possible partners of nuclear eEF1Bγ were involved to different neurodegenerative disorders (Kapustian et al., 2018; Kapustian et al., 2019). One cannot exclude that the specific localization of subunits may be associated with these diseases. The potential involvement of eEF1B in neurodevelopmental disorders was recently reviewed (McLachlan et al., 2019).

## Conclusion

There are two recently emerging directions of eEF1B complex studies. These are: (i) exploring the molecular mechanisms and regulatory consequences of the appearance of its individual subunits under abnormal cellular conditions; (ii) studying the links of different intracellular localization of the eEF1B complex and/or its individual subunits to various non-translational processes and, possibly, diseases. Combination of these approaches should be of interest for upcoming investigators to couple fundamental eEF1B knowledge with its evolving clinical significance.

## Author Contributions

The author confirms being the sole contributor of this work and has approved it for publication.

## Conflict of Interest

The author declares that the research was conducted in the absence of any commercial or financial relationships that could be construed as a potential conflict of interest.
